# A survival analysis of paediatric acute lymphoblastic leukaemia patients at the Moroccan University Hospital Centre of Rabat

**DOI:** 10.3332/ecancer.2025.1986

**Published:** 2025-09-15

**Authors:** Bennani Mechita Nada, Messaoud Sara, Elboukhari Elmamoun Yousra, Amina Kili, El Khorassani Mohammed, Lakhrissi Mariam, Isfaoun Zineb, El Ansari Naoual, Razine Rachid, Obtel Majdouline, El Kababri Maria, Hessissen Laila

**Affiliations:** 1 Laboratory of Community Health, Preventive Medicine, and Hygiene, Department of Public Health, Faculty of Medicine and Pharmacy, Mohammed V University in Rabat, Rabat 10100, Morocco; 2 Laboratory of Biostatistics, Clinical Research and Epidemiology, Department of Public Health, Faculty of Medicine and Pharmacy, Mohammed V University in Rabat, Rabat 10100, Morocco; 3 Moroccan Society of Paediatric Hematology and Oncology, Rabat 1005, Morocco; 4 Faculty of Medicine and Pharmacy, Mohammed V University in Rabat, Rabat 10100, Morocco; 5 Paediatric Hematology and Oncology Department, Children Hospital of Rabat, Mohammed V University, Rabat 1005, Morocco

**Keywords:** acute lymphoblastic leukaemia, paediatric, survival, Morocco

## Abstract

**Introduction:**

Acute lymphoblastic leukaemia (ALL) is the most common childhood leukaemia and a significant cause of paediatric mortality worldwide. Morocco, as part of the World Health Organisation (WHO) Global Initiative for Childhood Cancer, aims to achieve a 60% survival rate for paediatric cancers by 2030.

**Objective:**

This study evaluates survival rates and prognostic factors for paediatric ALL patients treated according to the MARALL 2006 protocol at the University Hospital Centre Ibn Sina in Rabat, Morocco.

**Methods:**

A retrospective cohort study analysed data from 512 children diagnosed with ALL between June 2006 and December 2017. Sociodemographic, clinical and therapeutic data were collected. Kaplan–Meier and Cox regression analyses identified survival rates and prognostic factors.

**Results:**

Among the patients, 56.2% achieved complete remission after first-line treatment and 20.9% experienced relapse. The 1-, 3- and 5-year overall survival rates were 83%, 67% and 63%, respectively. Significant prognostic factors included age ≥10 years, white blood cell count >50,000/mm^3^ and elevated lactate dehydrogenase levels. Standard risk classification and B-cell immunophenotype were associated with better survival outcomes.

**Conclusion:**

This study highlights encouraging survival rates for paediatric ALL patients in Morocco, exceeding the WHO target of 60%. However, achieving the national goal of 80% survival requires further improvements in early diagnosis, treatment access and adoption of advanced therapies.

## Introduction

Acute lymphoblastic leukaemia (ALL) stands as the predominant childhood leukaemia, representing 75% to 80% of all cases in children [1]. ALL is a hematological condition characterised by malignant proliferation of hematopoietic tissue, leading to bone marrow invasion by immature T or B lymphoid cells.

Morocco is a target country of the World Health Organisation (WHO) Global Initiative for Childhood Cancer (GICC), which was initiated in September 2018 to combat childhood cancer. The initiative aims to achieve a 60% 5-year survival rate for childhood cancer by 2030, through strengthened diagnosis, treatment and supportive care systems [2]. ALL is one of the six index childhood cancers that form the basis of this initiative.

According to the collaborative research program ‘Children's Cancers,’ approximately 800 new cases of paediatric cancers are diagnosed in Morocco each year [3]. Morocco has made significant progress in childhood cancer survival, currently achieving a rate of 60% due to the collective efforts of the country's various paediatric oncology services. A study conducted at the Paediatric Hematology and Oncology Department of the University Hospital Centre (UHC) Ibn Sina in Rabat between January 2012 and December 2014 concluded that ALL was the most frequent malignant hematological disease (74%), followed by acute myeloid leukaemia (20%) [4].

Morocco has launched a new national plan to combat childhood cancer, aiming to improve the survival rate to 80%. This plan was developed in close collaboration between the Ministry of Health and the Lalla Salma Foundation for Cancer Research and Prevention, under the initiative of the WHO [5]. Achieving this target will depend on further improving the survival of patients diagnosed with ALL.

Globally, the treatment of ALL has made significant progress in recent years, with a 5-year overall survival (OS) rate of approximately 90% [6]. A recent study conducted at six Paediatric Hematology and Oncology units in Morocco as part of the WHO GICC concluded that the 3-year OS rate for ALL was 68.2%, demonstrating that the standardised national approach based on the MARALL 2006 protocol notably improved the management of ALL [3, 7] in the country.

The aim of our study was to describe the survival rate and various prognostic factors that determine the outcomes of ALL among children treated with the national protocol MARALL 2006 at the Paediatric Oncology Department of UHC Ibn Sina in Rabat.

## Materials and methods

### Study type and population

This is a single-centre retrospective cohort study including children diagnosed with ALL over an 11.5-year period from June 2006 to December 2017 at the Paediatric Oncology Department of UHC Ibn Sina in Rabat.

### Eligibility criteria

This study included all children from the paediatric hematology and oncology centre aged between 1 and 15 years diagnosed with ALL based on a morphological study with or without immunophenotyping and treated according to the MARALL 06 protocol.

The initial diagnosis of ALL relied on the detection of ≥20% lymphoblasts in Wright-stained bone marrow smears. Immunophenotyping was then employed to validate the diagnosis according to WHO guidelines [8].

**Figure 1.**
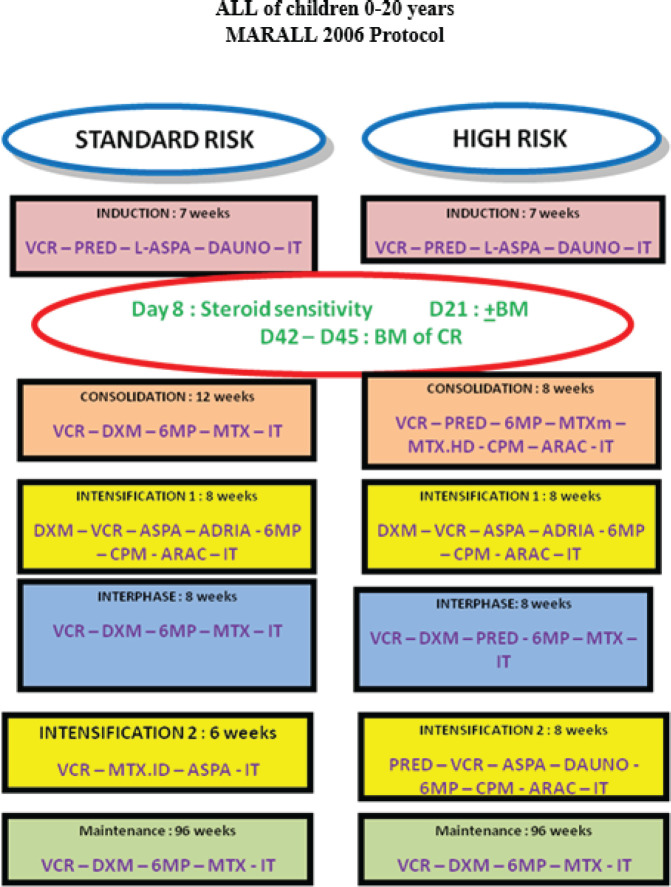
MARALL 2006 protocol.

Secondary ALLs, ALL in patients with Down syndrome, Philadelphia chromosome-positive ALL with t(9;22), patients transferred to another oncology centre before starting treatment and patients whose parents refused to initiate or continue treatment or who had received prior antineoplastic chemotherapy were excluded, according to MARALL 2006 protocol (Figure 1) [9].

### Data collection method

Data were collected from secondary sources, including existing databases of the paediatric oncology department. When data were incomplete, patient medical records were consulted. When the last contact date was far from the endpoint date, vital status data were collected from parents via phone calls. The data extraction forms included sociodemographic data, clinical, paraclinical, histological, therapeutic and follow-up data.

Remission status was assessed on day 42 of treatment for both risk groups, after hematologic recovery from aplasia. Complete morphological remission was defined as the absence of clinical signs, normalisation of peripheral blood counts and a cellular bone marrow containing less than 5% blasts. Minimal residual disease was not evaluated in this study, as the required techniques are currently unavailable in our setting [10].

Relapse was defined as either early or late based on the time from first complete remission (CR1). Early relapse was defined as occurring within 24 months after CR1 and late relapse was defined as occurring after 24 months. Relapse risk stratification was managed on a case-by-case basis. T-cell ALL relapses were systematically considered high-risk (HR), while B-cell relapses were classified according to the criteria outlined in the COPRALL 97 protocol, which takes into account factors such as site of relapse, time since CR1 and response to reinduction therapy [11].

The tumour syndrome corresponds to adenopathy, splenomegaly or any other lymphoid or extra-hematopoietic organomegaly [12].

The hemorrhagic syndrome corresponds to any type of hemorrhage; it includes purpura, ecchymoses or external hemorrhage such as gingival bleeding [13].

The patient inclusion period extended from June 2006 to December 2017. The study endpoint for data analysis was set as 30 November 2023, corresponding to the latest date on which follow-up information was collected.

The study start date was defined as the date of cytological diagnosis of ALL.

Patients were classified into HR and standard risk (SR) categories based on clinical and biological criteria according to MARALL’s classification (Table 1) at the time of diagnosis [14].

The two treatment groups, SR and HR, were defined as follows: SR patients were those aged between 1 and 10 years, with a white blood cell (WBC) count of less than 50,000/mm^3^, no central nervous system (CNS) involvement and a B-cell lineage. HR patients were those aged 10 years or older, with a WBC count of 50,000/mm^3^ or more, CNS involvement or a T-cell lineage. Patients classified as SR or HR received treatment according to the MARALL 06 protocol, which includes adapted therapeutic strategies based on age, WBC count, CNS involvement and immunophenotype.

Treatment included induction, consolidation, intensification and interphase courses (11 months) followed by maintenance (2 years). Chemotherapy consisted of Vincristine, Doxorubicin, L-Asparaiginase, corticosteroids, high and low-dose Methotrexate, Cyclophosphamide, Cytarabine, 6-mercaptopurine and 18 doses of triple intrathecal therapy [9].

### Statistical management and analysis

A descriptive analysis was initially performed, where quantitative variables are represented by mean +/- SD and qualitative variables by count and percentage. Then, an OS and an event-free survival (EFS), were conducted. The Kaplan–Meier method was used to estimate survival rates, the log-rank test to compare survival curves between different classes and the Cox model to identify prognostic factors. A significance threshold of 0.05 was set at the beginning. The analysis was done using Jamovi software version 2.3.18.

### Ethical consideration

Ethical aspects were considered. The protocol was approved by the ethics committee of the Faculty of Medicine and Pharmacy of Rabat under reference CERB 65-24. Furthermore, anonymity was respected for each study participant and all dataset were anonymised before analysis. No funding was received for this study.

## Results

### Descriptive analysis

We collected 630 cases of ALL, 512 were included in the study and 118 were excluded based on eligibility criteria. The sociodemographic data of the study sample can be seen in Table 2.

The median age of our population was 5.5 years with an interquartile range of (3; 9.75) years. We observed a predominance in the age group between 1 and 5 years, representing 49.6% (*n* = 254) of our sample. More than half (*n* = 327; 64%) of the patients were of urban origin. Most of our sample (*n* = 381; 74.6%) had medical coverage under the RAMED.

Most of our patients presented with a febrile syndrome, accounting for 66.5% (*n* = 337), followed by a tumour syndrome in 63.2% (*n* = 265) (Table 3). However, symptoms of CNS involvement were diagnosed in only 1.4% of patients. Furthermore, 349 patients were diagnosed with ALL type B, while type T was observed in 106 patients and mediastinal masses were present in 15.7% (*n* = 78).

After classifying our population according to MARALL criteria, we found that 57.2% of patients were HR, while 42.8% were SR.

### Management outcomes

**Figure 2.**
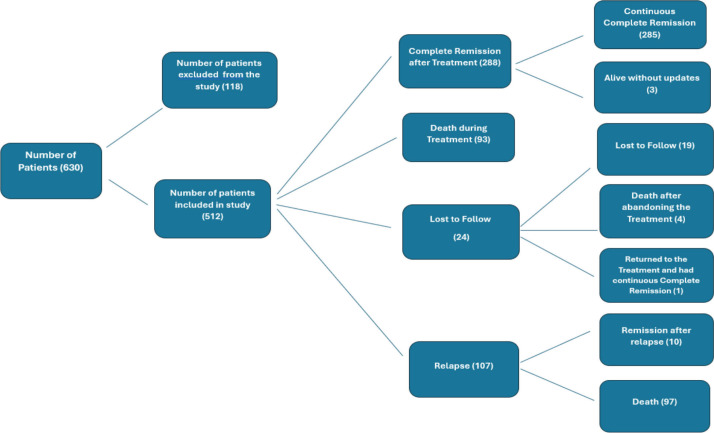
Diagram showing patient outcomes after treatment induction.

Twenty-four (4.6%) patients abandoned treatment after induction, 288 (56.2%) achieved complete remission after first-line treatment and 107 (20.9%) patients relapsed. Among those who relapsed, the majority (*n* = 75, 70%) were classified as HR, while the remaining relapsed patients (*n* = 32, 30%) were classified as SR (Figure 2).

Among the 190 deaths, 93 (49%) occurred during first-line treatment (toxic deaths) (Figure 2).

Among the deaths with available data, most occurred at home (62.6%), while 36.8% occurred in a hospital. The most frequent causes of death were infections (47.2%) and hemorrhagic complications (18.4%), followed by respiratory failure (5.6%), leukostasis (4.5%), renal failure (2.7%), treatment-related toxicity (4.0%), gastrointestinal complications (4.8%), metabolic disorders (0.8%), cardiovascular events (1.1%) and accidents (1.6%). Some patients had multiple contributing causes.

Regarding the timing of death after treatment initiation, 93 patients died during treatment. An additional 97 patients after relapse and 4 died after abandoning the treatment.

### Survival analysis

**Figure 3.**
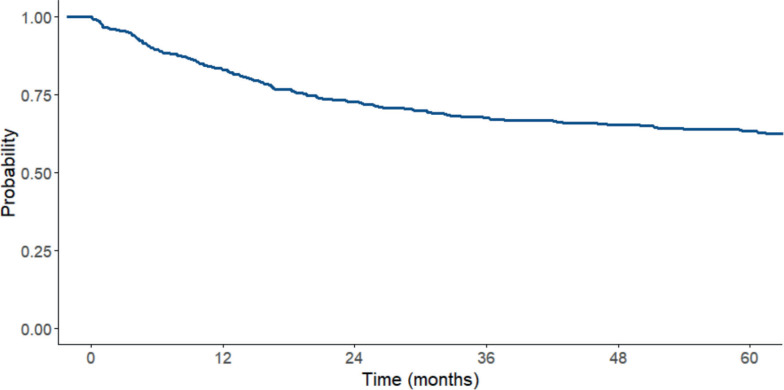
OS curve for the total ALL sample between 2006 and 2017.

Patients were followed from the date of diagnosis to the occurrence of an event or until the study endpoint. The overall median follow-up time, including both deceased and surviving patients, was 63 months (16.5–91.7). However, for patients alive at the end of follow-up, the mean duration of follow-up was 83.1 ± 30.6 months (Table 4). The complete remission rate was 56.2%. The 5-year OS rate was 63% for the entire cohort. It is important to note that among patients who did not achieve remission, the 5-year survival rate was 12.8% (Figure 3).

To explore potential improvements in survival over time, we divided the cohort into two groups based on the date of diagnosis: 2006–2011 and 2012–2017. The 5-year OS significantly increased from 58.9% (95% CI: 53.7%–64.6%) in the earlier period to 70.9% (95% CI: 64.6%–77.9%) in the later period. This difference was statistically significant (log-rank test, *p* = 0.0029), suggesting improved outcomes over the study period.

The 1-year survival rate was 83%, at 3 years it was 67% and at 5 years it was 63%. Children under 10 years old had higher survival rates (83.9% survival at 1 year and 65.7% at 5 years) compared to children over 10 years old (79.3% at 1 year and 53.2% at 5 years) (*p* = 0.028). Additionally, the survival rate among patients with WBC counts less than 50,000 cells/mm^3^ was higher (85% at 1 year and 67.5% at 5 years) compared to patients with WBC counts greater than 50,000 cells/mm^3^ (78.5% at 1 year and 53% at 5 years) (*p* < 0.001), also children with lactate dehydrogenase (LDH) ≤747 U/L (this cut off was chosen based on the median observed) had better survival than those with LDH>747 U/L (respectively, 73.4% and 58.8% 5 year survival, *p* = 0.005).

Furthermore, children classified as SR and those diagnosed with ALL type B had a better survival rate (*p* = 0.002).

In the multivariate Cox regression model, age group, maternal education and WBC group were statistically significant (*p* = 0.025, *p* = 0.020 and *p* = 0.008, respectively) (Table 5).

## Discussion

This study analysed the survival outcomes of children with ALL treated according to the MARALL 2006 protocol over an 11.5-year period at the Paediatric Oncology Department of Ibn Sina University Hospital in Rabat. The 5-year OS rate was 63%, exceeding the WHO GICC target of 60% but remaining below Morocco’s national goal of 80% by 2030 [5, 15].

The median age of patients was 5.5 years, with a predominance of the 1–5 years age group and a male-to-female ratio of 1.36, in line with studies from North Macedonia and Egypt [16, 17].

Immunophenotyping showed a predominance of B-cell ALL (76.7%) over T-cell (23.3%), similar to findings from North Macedonia [18]. Risk stratification revealed that 57% of patients were classified as HR, comparable to the Egyptian cohort [17].

Chemotherapy is the standard treatment for ALL administered in three phases (induction, consolidation and maintenance) over 2 to 3 years [19]. In our study, all patients underwent polychemotherapy according to the MARALL 2006 protocol. In developed countries, the complete remission rate is around 80% [20], whereas in our study, it was 56.2%, reflecting a notable disparity. In contrast, a study conducted in Egypt reported a remission rate of 93.1% [17]. Despite this lower remission rate, one of the key findings of our study was a significant improvement in OS over time. Patients diagnosed between 2012 and 2017 had better survival outcomes than those diagnosed between 2006 and 2011 (log-rank *p* = 0.0029). This encouraging trend may be explained by several factors: the introduction of structured training programs for healthcare personnel, the assignment of a dedicated nurse for leukaemia outpatient care and the gradual improvement of supportive care practices.

Among prognostic factors, survival was significantly lower in patients aged over 10 years and in those with WBC ≥50,000/mm^3^ at diagnosis. These findings confirm known risk factors reported in other studies, such as the ones conducted in Brazil and Egypt [17, 21].

The survival rates observed in our study (67% at 3 years and 63% at 5 years) are comparable to those reported in Ardabil, Iran [22], but still below the levels achieved in countries benefiting from access to targeted therapies and advanced supportive care [23].

In our study, the leading causes of death were infections and bleeding, accounting for 47.2% and 18.4% of cases, respectively. Similar trends have been reported in other resource-limited settings, such as in Egypt, where a study found that nearly a quarter of induction deaths were infection-related [24]. Hemorrhagic deaths, although less frequent, remain a major concern in paediatric hematologic malignancies, particularly in the context of thrombocytopenia and intensive chemotherapy [25].

The main causes of treatment failure in our study were relapse and toxic deaths. It is, therefore, more realistic for us to focus on improving survival by reducing toxicity-related mortality through enhanced supportive care. This includes improved hand hygiene, nurse and physician training, the implementation of quality improvement programs, access to supportive care resources (e.g., blood banks and dialysis) and better management of chemotherapy-specific toxicities such as mucositis and pancreatitis [26].

In a subsequent phase, the implementation of hematopoietic stem cell transplantation for selected relapsed cases could be considered. Beyond its direct therapeutic benefit, the development of a transplant program would foster a more structured, multidisciplinary approach to managing complex oncologic cases and contribute to raising the overall quality and standardisation of care [26].

To meet the national survival target of 80%, efforts must also include family support for transportation and nutrition, as well as the establishment of long-term follow-up programs. These should be complemented by psychological and social support for families, continuous training for healthcare workers and national research efforts on innovative treatments [27].

## Conclusion

In conclusion, this study reveals both strengths and challenges in the management of ALL in Morocco. We observed an encouraging complete remission rate of 56.2%. However, despite this, the 5-year OS rate of 63% remains below the desired 80% international benchmarks. This gap is significantly influenced by the low 5-year survival rate of 12.8% observed in patients who do not achieve remission. Our findings clearly indicate that further targeted efforts are essential to improve and elevate the OS rates.

## List of abreviations

ALL, Acute lymphoblastic leukaemia; CAR, Chimeric antigen receptor; CNS, Central nervous system symptoms; CR, Complete remission; EFS, Event-free survival; GICC, Global initiative for childhood cancer; HR, High risk; LDH, Lactate dehydrogenase; OS, Overall survival; RAMED, A medical assistance plan in Morocco: Régime d’assistance médicale; SR, Standard risk; UHC, University Hospital Centre; WHO, World Health Organization.

## Conflicts of interest

All authors declare no conflicts of interest.

## Funding

This study received no financial support or funding from any organisation.

## Author contributions

Bennani Mechita Nada, contributed to study design data analysis, manuscript writing and final revision

Messaoud Sara contributed to data analysis, manuscript writing and final revision.

Amina Kili, El Kababri Maria and Hessissen Laila contributed to the conception and study design, as well as the writing and final revision of the manuscript.

Elboukhari Elmamoun Yousra participated in data collection and manuscript writing

El Khorassani Mohammed, Lakhrissi Mariam, Isfaoun Zineb, El Ansari Naoual, contributed to study design, as well as the writing and final revision of the manuscript.

Obtel Majdouline and Razine Rachid participated in manuscript writing and final revision.

All authors have read and approved the final version of the manuscript.

## Figures and Tables

**Table 1.** Risk group criteria (MARALL 2006 Protocol).

	SR	HR
Age (years)	≥1 and <10	≥10
WBC (/mm^3^)	<50,000	≥50,000
CNS involvement	No	Yes
Lineage	B	T

**Table 2.** Sociodemographic characteristics of our population.

Characteristic	*N* (%)
**Age (years): (*n* = 511)** ** ≤5 years** **<10 years** ** ≥10 years**	254 (49.7) 130 (25.4) 127 (24.9)
**Sex: (*n* = 506)** **Female** **Male**	214 (42.0) 292 (58.0)
**Region of housing: (*n* = 510)** Urban **Rural**	327 (64.0) 183 (36.0)
**Medical coverage (*n* = 511)** **Mutual** **RAMED** **Without**	122 (23.9) 381 (74.6) 8 (01.6)
**Child’s education level: (*n* = 210)** **Primary** **Secondary** **Illiterate**	161 (76.6) 45 (21.4) 4 (01.9)
**Father’s education level: (*n* = 499)** **Primary** **Secondary** **Illiterate** **Tertiary**	119 (23.8) 112 (22.4) 226 (45.3) 42 (08.4)
**Mother’s education level: (*n* = 506)** **Primary** **Secondary** **Illiterate** **Tertiary**	83(16.4) 94 (18.6) 300 (59.3) 29 (05.7)
**Mother’s profession: (*n* = 508)** **Employed** **Unemployed**	27 (05.3) 481 (94.7)
**Father’s education: (*n* = 504)** Employed **Unemployed**	500 (99.2) 4 (00.8)

**Table 3.** Clinical and paraclinical characteristics.

Charateristics	*N* (%)
**Fever (*n* = 507)**	**337 (66.5)**
**Hemorrhagic syndrome (*n* = 496)**	**212 (42.7)**
**Tumor syndrome (*n* = 419)**	**265 (63.2)**
**Mediastinal mass (*n* = 498)**	**78 (15.7)**
**CNS symptoms (*n* = 504)**	**7 (01.4)**
**Tumor lysis syndrome (*n* = 232)**	**12 (05.2)**
**Initial assessment (WBC count) (*n* = 512)** ≤50,000/**mm^3^** **>50**,000/**mm^3^**	**17**,**400** (**5**,**723; 67**,**325**)* 357 (69.7) **155 (30**.**3)**
**Initial assessment (hemoglobin) (g/d**L**) (*n* = 512)**	**6.6500** (**4**.**97; 86**,**000**)
**Initial assessment (platelets) (/mm^3^) (*n* = 512)**	**39**,**000** (**18**,**450; 9**,**5325**)
**Initial assessment (LDH) (U/L) (*n* = 383)**	**747** (**381; 1**,**549**)
**Initial assessment (blasts in the blood) (*n* = 406)**	**63%** (**22%; 85%**)
**Initial assessment (blasts in the bone marrow) (*n* = 484)**	**90%** (**82%; 94%**)
**Immunophenotyping (*n* = 455)** **B-cell** **T-cell**	**349 (76.7)** **106 (23.3)**
**Risk according to MARALL criteria (*n* = 509)** **HR** **SR**	**291 (57.2)** **218 (42.8)**

* Median (interquartile range)

**Table 4.** Survival study of patients diagnosed with ALL.

Characteristic	1-year survival (%)	3-year survival (%)	5-year survival (%)	*p*-value
**OS**	83.0	67.0	63.0	x
**EFS: (relaps, progression)**	79.2	63.3	56.9	x
**Age (years)** 1–10 years >10 years	83.9 79.3	69.8 58.8	65.7 53.2	0.028
**Sex** **Female** **Male**	80.7 84.7	67.7 67.7	63.9 63.0	0.880
**WBCs** ≤50,000**/mm^3^** >50,000**/mm^3^**	85.0 78.5	71.6 58.8	67.9 53.0	<0.001
**LDH level (U/L)** ≤**747** **>747**	87.4 81.5	77.1 63.3	73.4 58.8	0.005
**Risk according to MARALL criteria** **HR** **SR**	80.3 87.0	63.3 73.8	58.0 70.8	0.002
**Immunophenotyping** B-cell T-cell	84.9 74.1	71.0 54.7	66.6 50.5	0.002

**Table 5.** Univariate and multivariate analysis using Cox regression model.

Characteristics	Univariate analysis		Multivariate analysis	
	HR (95%) IC	*p*	HR (95%) IC	*p*
**Age (Years)** 1–**10 years** ≥**10 years**	1.00 1.44 (1.04;2.00)	0.003	1.59 (1.06;2.39)	0.025
**Sex** Female Male	1.00 0.89 (1.73;1.30)	0.880	0.97 (0.67;1.42)	0.892
**Mother’s education** Primary Illiterate Secondary Tertiary	1.00 0.92 (0.64;1.33) 0.40 (0.23;0.69) 0.76 (0.39;1.49)	0.659 0.001 0.426	0.82 (0.52;1.31) 0.47 (0.25;0.89) 0.83 (0.38;1.84)	0.412 0.020 0.654
**Immunophenotyping** **B-cell** **T-c**ell	1.00 1.65 (1.19;2.28)	0.002	1.13 (0.73;1.74)	0.598
**WBCs** ≤50,000/mm**^3^** >50,000/mm**^3^**	1.00 1.70 (1.28;2.27)	<0.001	1.73 (1.16;2.57)	0.008
**LDH** ≤747 UI/L >747 UI/L	1.00 1.63 (1.15;2.30)	0.006	1.31 (0.88;1.94)	0.188
